# Nasal and cutaneous mucormycosis in two patients with lymphoma after chemotherapy and target therapy: Early detection by metagenomic next-generation sequencing

**DOI:** 10.3389/fcimb.2022.960766

**Published:** 2022-09-16

**Authors:** Qing Zhang, Xingchen Liu, Yanyan Liu, Huiqin Wang, Ran Zhao, Xiaodong Lv, Xudong Wei, KeShu Zhou

**Affiliations:** Department of Hematology, The Affiliated Cancer Hospital of Zhengzhou University and Henan Cancer Hospital, Zhengzhou, China

**Keywords:** metagenomic next-generation sequencing, mucormycosis, nasal infection. *Lichtheimia ramosa*, *M. irregularis*, cutaneous infection, lymphoma

## Abstract

Mucormycosis is a conditionally pathogenic fungal disease with high morbidity that mainly affects patients with decreased immunity. Diagnosis relies on the histopathological examination of microorganisms with the typical structure of mucormycetes in tissues and subsequent confirmation *via* culture. Early detection of causative microorganisms is critical to rapidly administer appropriately targeted antibiotics. Metagenomic next-generation sequencing (mNGS) is an innovative and sensitive technique used to identify pathogenic strains. Here we used mNGS to timely diagnose an infection with *Lichtheimia ramosa* and *Mucor irregularis* in two patients with hematologic malignancies; the infections manifested as nasal and cutaneous infections and developed after chemotherapy and small molecule targeted therapy. Following treatment with amphotericin B cholesteryl sulfate complex, the symptoms were reduced significantly, and both patients obtained successful outcomes. Additionally, we searched and summarized the current medical literature on the successful diagnosis of mucormycosis using mNGS. These cases indicated that mNGS, a novel culture-independent method, is capable of rapid, sensitive, and accurate identification of pathogens. mNGS may be a complementary method for the early identification of mucormycosis, allowing for appropriate and timely antibiotic administration and thus improving patient outcomes.

## Introduction

Mucormycosis is an opportunistic fungal disease with high morbidity that primarily affects patients with decreased immunity. In the past two decades, it has become the third most prevalent invasive mold disease in patients with hematologic malignancies (HM), following candidiasis and aspergillosis (Cornely et al., 2019; Jeong et al., 2019). Pulmonary, rhino-orbital-cerebral, cutaneous, and disseminated are the most common clinical manifestations of mucormycosis (Vehreschild et al., 2013). Invasive mucormycosis is difficult to diagnose owing to nonspecific clinical manifestations and imaging (Skiada et al., 2020). Traditional microbiological identification methods include histopathology and culture; however, cultures are frequently negative and time-consuming, and biopsy may not always be viable for individuals with compromised immunity (Roden et al., 2005; Skiada et al., 2011). Owing to the rapid progression of mucormycosis, timely diagnosis and the administration of antifungal therapy are crucial to improve patient prognosis. Thus, there is an urgent clinical need for a molecular diagnostic tool that can reliably and efficiently identify pathogens and guide the treatment for patients.

Metagenomic next-generation sequencing (mNGS) is a new genomics-based pathogen detection method relying on a single run to obtain nucleic acid sequence information about microbes to detect microorganisms through analysis and comparison (Ghez et al., 2018). Moreover, mNGS can be used to identify all types of pathogens since it does not rely on culture and retrieves DNA without bias (Chamilos et al., 2008). mNGS has significant advantages over typical pathogen detection approaches, as it can rapidly identify pathogens that cannot be cultivated or are difficult to culture (Walsh et al., 2012). Recently, it has been developed to detect a wide range of microbial infections in patients with HM and in transplant recipients (Schwartz et al., 2018).

Here we present two cases of patients with lymphoma with nasal and cutaneous mucormycosis after chemotherapy and treatment with Bruton’s tyrosine kinase inhibitor. Initially, the clinical manifestation was mainly fever, and the empirical anti-infection effect was not satisfactory. The patients were diagnosed with *Lichtheimia ramosa* and *Mucor irregularis* infection *via* mNGS of peripheral blood, later confirmed as mucormycosis by secretory culture. Appropriate antibiotics were applied in the early stage, and the prognosis was good. This case report corresponds to the CARE guidelines (Limmathurotsakul et al., 2010).

## Case presentation

### Case 1

**Figure 1:**
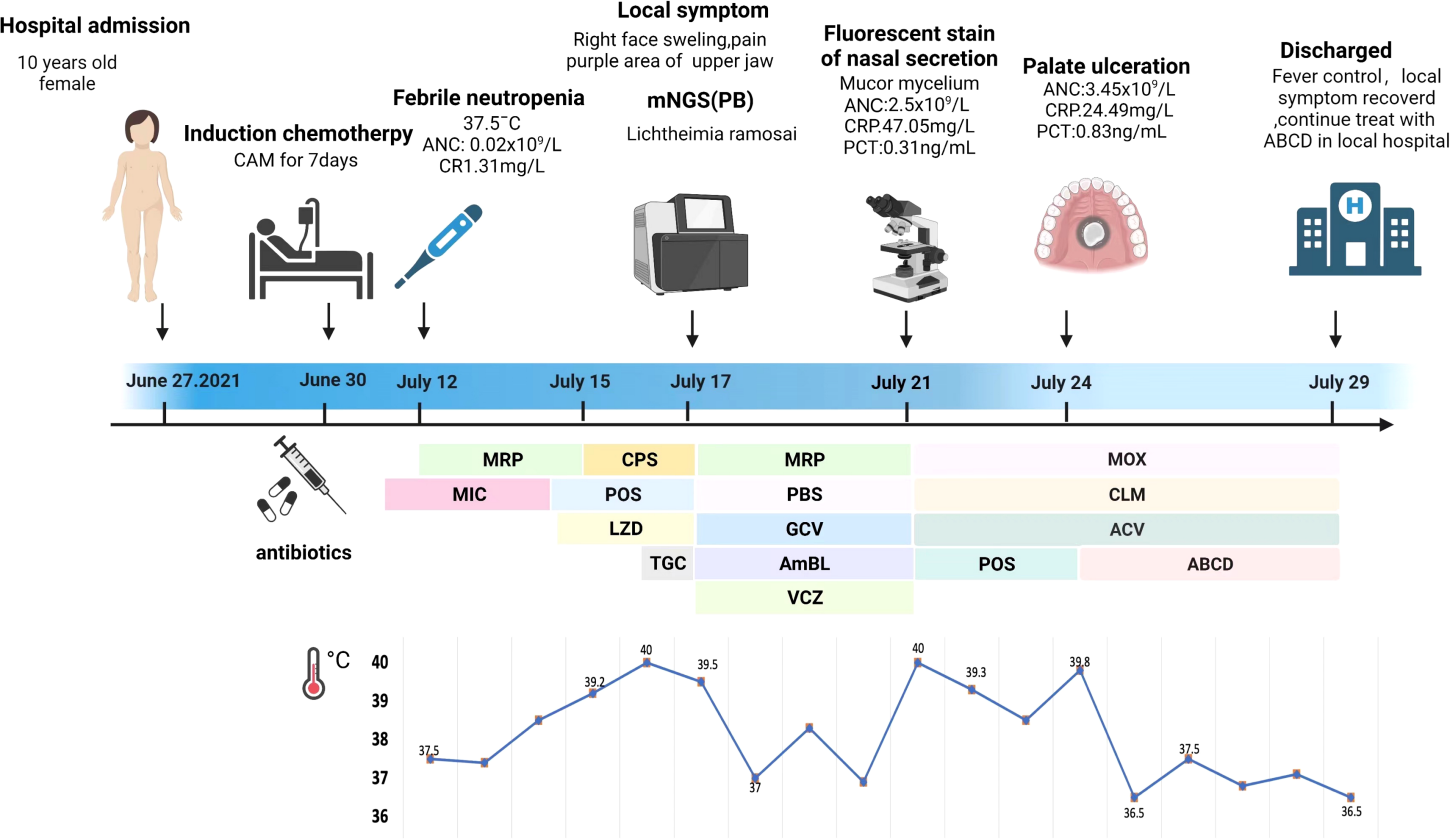
Episode timeline of case 1. CRP, C-reactive protein; PCT, procalcitonin; ANC, absolute neutrophil count; MRP, meropenem; MIC, micafungin; CPS, cefoperazone sulfate; POS, posaconazole; LZD, linazolamide; TGC, tigecycline; PBS, polymyxin B sulfate; GCV, ganciclovir; AmBL, amphotericin B liposome; VCZ, voriconazole; MOX, moxalactam; CLM, clindamycin; ACV, acyclovir; ABCD, amphotericin B cholesterol sulfate complex.

A 10-year-old girl with T-lymphoblastic lymphoma was admitted to our hospital on June 27, 2021. She underwent a third cycle of induction chemotherapy on June 30 for 7 days. A timeline of the episodes is shown in **Figure 1**.

The patient reported a mild cough 3 days later. Micafungin (50 mg daily, intravenously—IV) was administered as the primary antifungal prophylaxis; the patient’s symptoms improved after the treatment. Bone marrow suppression began on July 10 and lasted for 11 days; colony-stimulating factor was added as supportive care. Febrile neutropenia occurred 2 days later, with the patient having a body temperature of 37.5°C. The white blood cell count was 0.31 × 10^9^/L according to the blood routine examination. The absolute neutrophil count (ANC) was 0.02 × 10^9^/L, and the C-reactive protein (CRP) level reached 1.31 mg/L. Immediate empirical antibiotic therapy with meropenem (MRP, 0.5 q/8 h, IV) was administered, and her fever subsided for 2 days.

On July 14, the body temperature climbed to 38.6°C, and the blood routine examination showed that the ANC was 0.02 × 10^9^/L and the procalcitonin (PCT) level was 0.6 ng/ml. Linazolamide (LZD) (300 mg/8 h, IV) was added to treat Gram-positive cocci, and the antifungal treatment was modified to posaconazole suspension (POS, 5 ml/6 h, orally). However, the body temperature was not controlled. On July 15, the temperature rose to 39.5°C and was accompanied with shivers. The ANC had risen to 0.03 × 10^9^/L, the PCT level had risen to 3.23 ng/ml, and the CRP level was 44 mg/L. MRP was replaced with cefoperazone sulfate (1.5 g/8 h, IV) coupled with LZD as a broad-spectrum antibacterial treatment. High-grade continuous fever with chill still remained. At that time, a series of cultures of peripheral blood were negative and no pathogen could be detected. The (1,3)-β-D glucan and galactomannan antigen tests were negative, and no obvious abnormalities were observed in the chest computed tomography (CT) images. Considering a history of abdominal infection after the last cycle of chemotherapy, tigecycline (40 mg/12 h, IV) was used to resist an abdominal complex bacterial infection on July 16.

**Figure 2:**
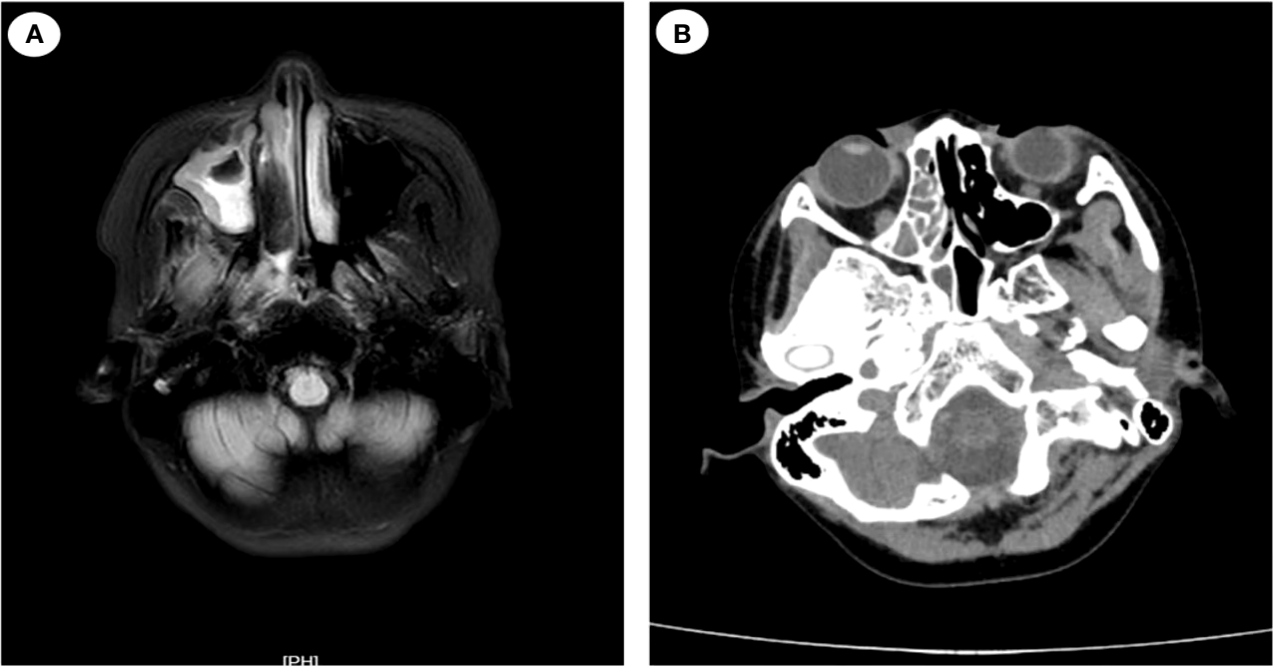
Magnetic resonance imaging (MRI) and computed tomography (CT) images of the patient’s head (case 1). **(A)** MRI of the patient’s head on July 17: abnormal signal shadow of the ethmoid sinus, sphenoid sinus, and bilateral maxillary sinus. **(B)** CT scans of nasopharynx on July 24: soft tissue shadows are observed in the right nasopharynx, ethmoid sinus, nasal cavity, and maxillary sinus.

On July 17, the patient complained of severe pain on the left side of her face as well as had a high temperature and chills. The physical examination revealed left facial swelling and restricted expression; the left nasolabial groove was shallow, and a bluish-purple patch could be seen in the palate. The magnetic resonance imaging results of the patient’s brain showed abnormal signal shadows of the ethmoid sinus, sphenoid sinus, and bilateral maxillary sinus, considering sinus infections (**Figure 2**). The treatment was upgraded to polymyxin B sulfate (250,000 IU/12 h, IV) to cover multidrug-resistant negative bacteria immediately. Subsequently, mNGS (MGISEQ-2000 sequencing platform; MGI, Shenzhen, China) of peripheral blood detected *L. ramosa* with high relative abundance as well as simplex virus and pegivirus (**Table 1**). Amphotericin B liposome (AmBL, 30 mg daily, IV) combined with voriconazole (VCZ, 0.1 g/12 h) was immediately used as a broad-spectrum antifungal therapy. Ganciclovir (0.15 g/12 h, IV) was administered as antiviral treatment alongside supportive care, such as intravenous hyper-nutrition. From July 17 to 20, the patient’s fever interval was prolonged, and the heat peak was lower. However, the facial edema did not diminish considerably.

**Table 1:** Literature review: Cases of mucormycosis diagnosed with metagenomic next-generation sequencing.

Case number	Sample		Genus		Species	
1	PB	Type	Name	Sequence number	Name	Sequence number
		Fungus	*Lichtheimia*	3414	*Lichtheimia ramosa*	3330
		Virus	Simplex virus	1	Human alphaherpevirus	1
			Pegivirus	466	Pegivirus C	466
2	PB	Fungus	Mucor	55	Mucor irregularis	55
		Virus	Cytomegalovirus	1031	Human betaherpesvirus (CMV)	1022
			Alphatorquevirus	998	Torque teno virus 19	760
			Simplex virus	6	Human alphaherpesvirus	6

PB, peripheral blood.

On July 21, severe fever returned, with a *T*_max_ of 40°C. Her ANC recovered from neutropenia at that time. Infection with Mucorales was indicated by fluorescent staining of nasal secretions, which revealed wide, pauci-septate hyphae. The AmBL and VCZ combination was replaced with POS injection as antifungal treatment. The antiviral treatment was changed to moxalactam (1 g/12 h, IV) and clindamycin (0.3 g/12 h, IV) combined with acyclovir (0.25 g/8 h, IV), and amphotericin B atomization inhalation was used to relieve local symptoms. However, intermittent fever persisted during the treatment, soft tissue infection rapidly progressed, mucosal necrosis was observed, and a defect in the upper jaw occurred, accompanied by bone pallor. The CT scans of the nasopharynx showed soft tissue shadows in the right nasopharynx, ethmoid sinus, nasal cavity, and maxillary sinus; fortunately, bone destruction was not found (**Figure 2**). After pediatric head and neck surgery, a multidisciplinary consultation with the pharmacy department diagnosed the patient with nasal mucormycosis. Amphotericin B cholesterol sulfate complex (ABCD) (2 to 3 mg/kg daily, IV) was suggested, combined with amphotericin B local nasal drip and debridement of the nasal sinuses after systemic infection control. The scope of the lesions was greatly decreased, the local symptoms were relieved, and the body temperature was stabilized after 2 days of treatment.

The patient was discharged and sent to a local hospital for ABCD anti-infection treatment for 3 weeks and POS suspension (5 ml/6 h, orally) for another 2 months. Imaging examination was repeated every subsequent month, and the nasal sinus infection did not spread any further. The antifungal treatment regimen was well tolerated, with no return of mucormycosis after follow-up by phone until February 2022.

### Case 2

**Figure 3:**
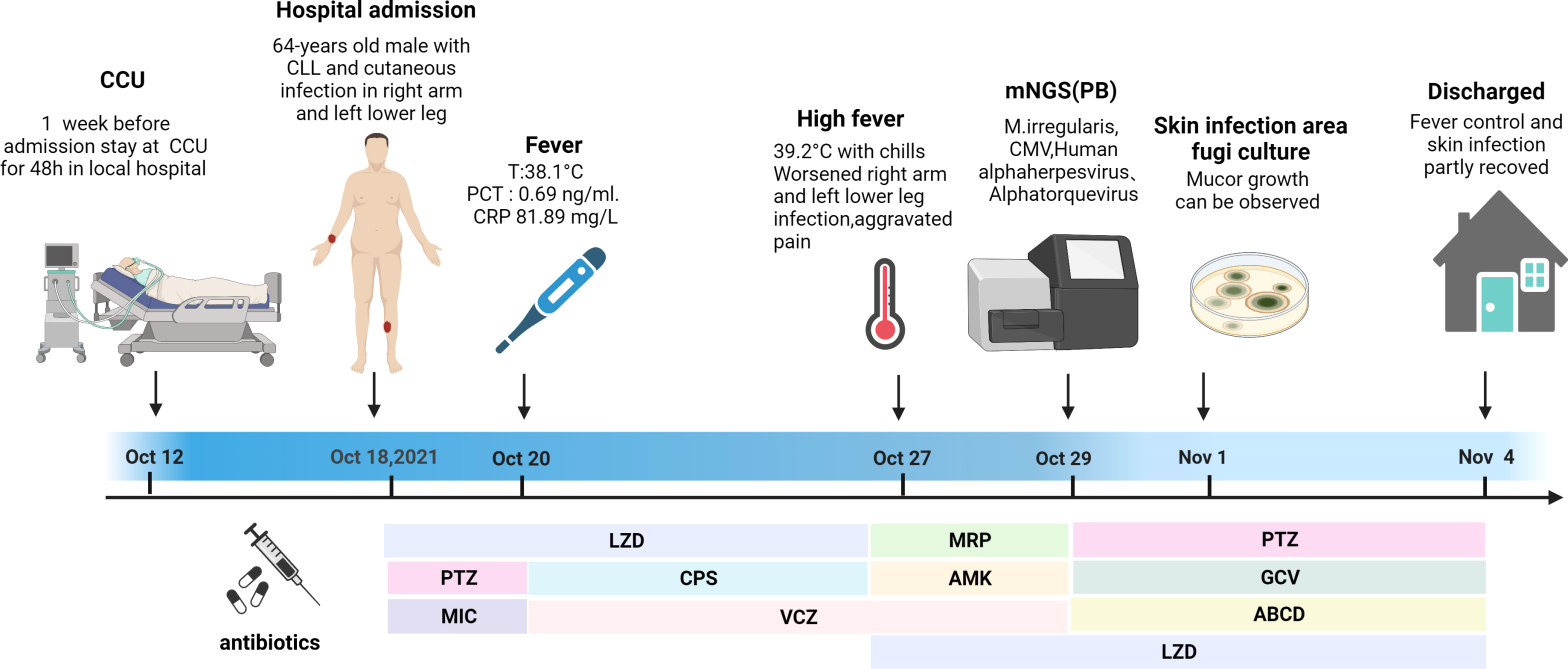
Episode timeline of case 2. CRP, C-reactive protein; PCT, procalcitonin; CCU, coronary heart disease care unit; LZD, linazolamide; PTZ, piperacillin sodium and tazobactam; MIC, micafungin; CPS, cefoperazone sulfate; VCZ, voriconazole; MRP, meropenem; AMK, amikacin sulfate; GCV, ganciclovir; ABCD, amphotericin B cholesterol sulfate complex.

A 64-year-old man suffering for 6 years from chronic lymphocytic leukemia (CLL) was admitted to our hospital on October 18, 2021. He had been continuously taking ibrutinib for CLL. At 1 week before admission, he was transported to the coronary heart disease care unit of the local hospital for a 48-h stay owing to a sudden acute myocardial infarction. He developed a skin infection after applying the restraint belt. **Figure 3** depicts a timeline of the episodes.

**Figure 4:**
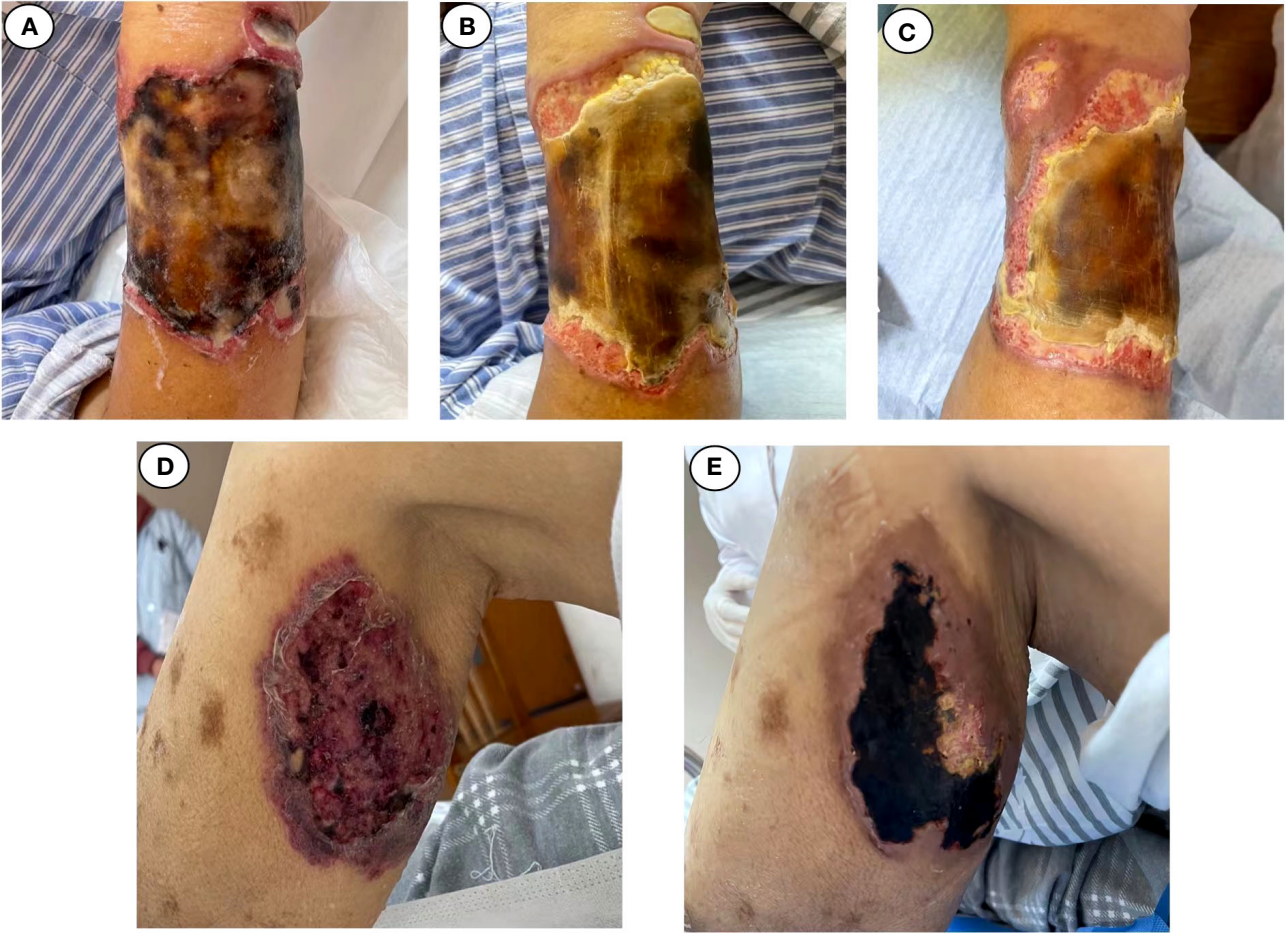
Changes in cutaneous fungal infection (case 2). **(A)** Admitted. **(B)** November 1. **(C)** November 4. **(D)** Admitted. **(E)** November 4.

Upon admission, the patient’s vital signs were normal. A physical examination revealed anemia, with a 15 × 20-cm eroded region on the right upper limb covered in black eschar as well as additional necrotic tissue and effusion. On the left lower leg, there was a 5 × 6-cm erosion surface (**Figure 4**). Both lower limbs had edema. Other physical examinations disclosed no obvious issues. The blood routine examination showed the following: white blood cell count—23.18 × 10^9^/L, ANC—21.15 × 10^9^/L, HGB—79 g/l, PLT—36 × 10^9^/L, and positive Coombs test. The PCT level was 1.3 ng/ml, and the CRP level was 99.78 mg/L. The (1,3)-β-D glucan test was positive, and the galactomannan antigen test was negative. The electrocardiogram revealed ST segment abnormalities as well as aberrant Q waves (II, III, and aVF). The chest CT result showed pulmonary infection. The color Doppler ultrasound showed thrombosis of the right basilar vein and left leg intermuscular vein. Based on these findings, CLL progression was diagnosed, along with hemolytic anemia, cutaneous infection, lung infection, a previous myocardial infarction, and venous thrombosis. An initial treatment of LZD (0.2 g/12 h, IV), micafungin (150 mg daily, IV), and piperacillin sodium and tazobactam (4.5 g/8 h, IV) was empirically administered. Ibrutinib was stopped, and fresh frozen plasma combined with rituximab and low-dose methylprednisolone were administered to control CLL. Meanwhile, component blood transfusion, anticoagulation, analgesia, organ function protection, and other symptomatic treatments were administered. Unfortunately, the patient was not completely relieved from his local problems.

The patient reported fever with a body temperature of 38.1°C. At 2 days later, the PCT level was 0.69 ng/ml, and the CRP level was 81.89 mg/L. To improve the etiological evaluation, his peripheral blood and foci secretions were analyzed to identify potential pathogens; however, they were all negative. The anti-infection treatment was altered to VCZ (0.2 g/12 h, IV), cefoperazone sulfate (3 g/8 h, IV), and LZD as empirical antifungal and antibacterial therapy. Although the body temperature was controlled, the skin infection symptoms did not improve.

On October 26, his temperature reached 39.2°C and was accompanied with shivering. Peripheral blood was drawn to test for pathogens *via* culture and mNGS. MRP (1 g/8 h, IV), amikacin sulfate (0.4 g/d, IV), and LZD were administered combined with VCZ as a broad-spectrum antimicrobial therapy.

Within 48 h, the mNGS results were released: *M. irregularis*, human betaherpesvirus, human alphaherpesvirus, and *Alphatorquevirus* were detected (**Table 1**). The subsequent fungal culture of the secretion revealed *Mucor* growth, confirming the cutaneous mucormycosis. The antibacterial treatment was initiated with LZD and piperacillin sodium and tazobactam. The antiviral treatment began with ganciclovir (0.375 g/12 h, IV), and the antifungal treatment was adjusted to ABCD (150 mg daily, IV). Local debridement and antifungal medication were also initiated. The body temperature was stabilized after 3 days of treatment, and the local symptoms were reduced dramatically (**Figure 4B**). With continuing therapy, the necrotic tissue progressively peeled off, and fresh granulation tissue formed (**Figures 4C, E**).

Due to financial concerns, the patient was discharged from the hospital on November 4 and transferred to a local hospital for anti-infection treatment. He was routinely contacted *via* telephone. The patient is taking venetoclax orally to treat CLL, he is in good health, and the skin wound has completely healed.

## Methods for mNGS

Blood samples (5 ml) were collected into EDTA tubes (Becton, Dickinson and Company, Franklin Lakes, NJ, USA) and centrifuged at 1,600 × *g* for 10 min. Cell-free DNA was extracted from the plasma supernatant. After centrifugation, 600 μl supernatant was removed, and DNA was extracted using a micro-sample genomic DNA extraction kit (1901; Genskey, Tianjin, China). Using an NGS library construction kit, the DNA libraries were constructed by DNA enzyme digestion (200–300 bp), end-repair, a-tailing, adapter ligation, and polymerase chain reaction (PCR) amplification (2012B; Genskey). Before sequencing, the DNA library quality was evaluated using an Agilent 2100 Bioanalyzer (Agilent Technologies, Santa Clara, CA, USA) in conjunction with qPCR to measure the adapters. Two to three quantitative sets of circular single-stranded DNA were added to create DNA nanospheres. The DNA nanospheres were placed onto the sequencing chip and sequenced on the MGISEQ-2000 device (MGI, Shenzhen, China). We employed an environment control sample (human nucleic acid) for each run to monitor microbial DNA signals originating from the background during batch processing as well as distinct ID spike variations (*Arabidopsis*-specific fragments) to monitor sample-to-sample contamination.

For quality control, adapter contamination, and low-quality and low-complexity reads, raw reads were filtered by fastp (v0.19.5) and Komplexity v0.3.6. Reads that were mapped to human reference assembly GRCh38 were removed with bowtie2 v2.3.4.3. The reads were then aligned to a microorganism database containing approximately 12,000 genomes using SNAP v1.0beta.18, as previously described. For each species, the mapped reads were categorized using either the National Center for Biotechnology Information RefSeq genome database or the GenBank genome database. We used Perl scripts to count the species or genus abundance after filtering out false-positive organisms.

For all pathogens originally detected, we first filtered out the obvious sequence alignment abnormalities (for the detected species, genome coverage <1% and depth <2) and background polluted bacteria (considered to be the exact background bacteria when the taxon-specific read number falls within the normal fluctuation range of historical statistical data compared with the negative controls). Pathogen data interpretation and pathogen-positive determination were then performed.

## Discussion

Mucormycosis is a difficult-to-diagnose fungal infection with high morbidity and mortality (Cornely et al., 2019). The most common pathogens are *Rhizopus* spp., *Mucor* spp., and *Lichtheimia* spp. (including *Absidia* spp. and *Mycocladus* spp.), accounting for 75% of all cases of mucormycosis (Vehreschild et al., 2013; Jeong et al., 2019). The next most common pathogens are *Rhizomucor* spp., *Apophysomyces* spp., *Saksenaea* spp., and *Cunninghamella* spp (Vehreschild et al., 2013).. Additionally, uncommon species such as *Synchephalastrum racemosum*, *Thamnostylum lucknowense*, *Cokeromyces* spp., and *Actinomucor elegans* have been reported since the advent of molecular techniques (Jeong et al., 2019). The most significant risk factors for mucormycosis are diabetes mellitus, HM, other cancers, ketoacidosis, hematopoietic stem cell transplantation, neutropenia, corticosteroids, trauma, iron overload, illicit intravenous drug use, neonatal prematurity, and malnutrition (Skiada et al., 2020). In people with normal immunity, trauma- or burn-related skin lesions may potentially get infected as well (Skiada et al., 2020).

Patients with HM and recipients of hematopoietic stem cell transplantation have the poorest prognosis (Roden et al., 2005). In recent decades, the incidence of mucormycosis has increased, primarily as a consequence of the increased number of patients with severe immunosuppression (Skiada et al., 2011). Herein we reported two patients with HM who developed severe nasal and cutaneous mucormycosis following chemotherapy and Bruton’s tyrosine kinase inhibitor (ibrutinib). According to ibrutinib-related disease association research, fungal infections are prevalent among patients with CLL and lymphoma (Roden et al., 2005). According to Ghez et al. (Ghez et al., 2018), the mechanism may involve a compromised innate/adaptive antifungal immune response during the initial phases of ibrutinib immunotherapy (during the first 6 months).

Mucormycosis is rapidly progressing and destructive, and thus delaying the initiation of therapy leads to increased mortality. A prior study in 70 patients with HM with mucormycosis demonstrated that more than 6 days of treatment delay doubled the mortality rate (Chamilos et al., 2008). Early diagnosis and rapid therapeutic intervention are of utmost importance because these maximize the survival rates and may also reduce the need for surgical excision, disfigurement, and suffering (Walsh et al., 2012). Diagnostic methods consist of risk factor identification, clinical manifestation evaluations, early utilization of imaging techniques, and timely initiation of diagnostic approaches based on histopathology, smear microscopy, culture (growth and isolation of microorganisms), and advanced molecular techniques such as microbial nucleic acid and antigen detection tests, serology (detection of pathogen-​specific antibodies), and exhaled breath metabolomics (Skiada et al., 2020). However, *Mucor* infection diagnosis is difficult in HM because of its nonspecific clinical manifestations; fever may be the only sign of infection. Pathogen detection is also challenging. Histopathology and routine culture are the gold standards, but biopsy is often difficult in patients with underlying HM (Schwartz et al., 2018). Moreover, owing to prior antibiotic and antifungal exposure, the sensitivity of routine culture is frequently low (Limmathurotsakul et al., 2010). In up to 50% of mucormycosis cases, the culture can be erroneously negative (Lackner et al., 2014). Additionally, histopathology and routine culture are time-consuming. Both patients described herein experienced such issues. The first patient that we reported had a recurrent high fever as the major manifestation for 5 days in the agranulocytosis phase. The repeated blood cultures were negative, facial edema developed later, imaging revealed atypical sinusitis, and the repeated empirical anti-infection adjustments were ineffective. Skin infection and fever were the predominant manifestations in the second patient, and secretory and blood cultures were negative at the beginning of the anti-infection treatment, resulting in an increased severity of symptoms and a challenging diagnosis.

Molecular-based methods such as PCR and mNGS lead to earlier diagnosis compared to culture (Millon et al., 2016). The amount of circulating Mucorales DNA in serum was shown to be particularly high compared to invasive aspergillosis, most likely due to the angio-invasive nature of Mucorales infection (Ibrahim et al., 2012; Millon et al., 2016). Attempts directed at molecular-based diagnosis from blood and serum have yielded promising clinical results (Millon et al., 2016). PCR is hypothesis-driven and requires prior knowledge of the suspected pathogen, and concerns have been raised regarding detection sensitivity; hence, it has only been used for preliminary screening (Gu et al., 2021). mNGS is a non-biased technology for quick pathogen detection that performs DNA or RNA sequencing directly from samples. Multiple specific pathogen tests can be replaced with a single mNGS assay, which enables the detection of a broad range of pathogens (viruses, bacteria, fungi, and parasites) directly from clinical samples without requiring doctors for prejudgment (Miao et al., 2018; Simner et al., 2018). Therefore, mNGS provides advantages over PCR for diagnosing and detecting certain rare or unknown infections. mNGS may thus be specifically relevant for the diagnosis of difficult-​to-diagnose cases or for immunocompromised patients in whom the spectrum of potential pathogens is increased (Chiu and Miller, 2019). In addition, mNGS can overcome the constraints of conventional culture, as it allows for the detection of known infections in 24–48 h, greatly shortening the diagnosis time of infectious pathogens (Miao et al., 2018; Chiu and Miller, 2019). Rapid reporting of mNGS results can also facilitate the timely modifications of therapy in clinical practice (Gosiewski et al., 2017). In our present cases, mNGS allowed for the rapid and accurate identification of pathogens, which were confirmed by subsequent local secretory culture results. When multiple broad-spectrum anti-infections fail, mNGS-based anti-mucormycosis treatment controls the infection.

The considerable potential of mNGS in mucormycosis diagnoses has been demonstrated by multiple successful cases and studies. Most published works are case reports. We list the most recent mNGS mucormycosis diagnosis reports in **Table 2**. All these cases indicate that mNGS can be used to diagnose rare infectious diseases with a variety of symptoms and in multiple sample types as follows:

**Table 2:** Literature review: Cases of mucormycosis diagnosed with metagenomic next-generation sequencing (mNGS).

Age and sex	Underlyingdiseases	Risk factors	Infection sites	mNGS sample	Pathogen	Other etiological detection	Reference
44 male	ALL	Bone marrow suppression	Disseminated Cerebral infarction	PB, BALF, CSF	*Rhizomucor miehei*	PB cultures are negative	(Wen et al., 2021)
62 male	CHB Diabetes Psoriatic arthritis	Trauma Open wounds	Disseminated, skin, lung	Skin debridement tissue, lung tissue	*Rhizopus microsporus*	Final pathology of the lung confirms a mucor infection	(Sun et al., 2021)
53 male	Healthy	Retrograde infection after tooth extraction	ROCM	CSF	*Lichtheimia ramosa*	Secretions, PB and CSF cultures are negative	(Liu et al., 2021)
68 male	Diabetic ketoacidosis	Uncontrolled blood sugar Broad-spectrum antibiotics	ROCM	PB, CSF	*Rhizopus arrhizus*	CSF qPCR verified by *R.* arrhizus	(Dong et al., 2022)
Male	T-ALL	Bone marrow suppression, uncontrolled leukemia	Lung, nasal skin	PB, local secretion	*Mucor racemosus*	PB cultures are negative; nasal secretion culture confirmed a mucor infection	(Cui et al., 2022)
18 male	Ph-like ALL	Neutropenia	Lung, skin, liver, kidney, spleen, brain	PB	*Rhizomucor pusillus*	Skin and liver lesions issue confirmed by qPCR analysis R pusillus	(Shi et al., 2022)
37 male	AFM, emphysematous gastritis	ECMO treatment	Gastrointestinal	PB	Mucormycosis	Pathology of the stomach showed mucor mycelium	(Wang et al., 2021)
12 male	ALL	Glucocorticoids Neutropenia	Lung, right rib	BALF	*Rhizopus oryzae*	NA	(Sun et al., 2022)
4 male	ALL	Neutropenia Broad-spectrum antibiotics	Disseminated, intracranial, right iliac bones, hip joints	BALF	*Rhizomucor pusillus*	BALF cultures are negative, fluorescent staining microscopy of BALF detects mucor mycelium	(Sun et al., 2022)
2-year-old female	ALL	Glucocorticoids Neutropenia	Lung	BALF	*Rhizomucor pusillus*	Fluorescent staining microscopy of BALF detects mucor mycelium	(Sun et al., 2022)

ALL, acute lymphoblastic leukemia; T-ALL, acute T lymphoblastic leukemia; Ph-like ALL, Philadelphia-like ALL; AFM, acute fulminant myocarditis; ROCM, rhino-orbito-cerebral; PB, peripheral blood; BALF, cerebral spinal fluid; CSF, cerebral spinal fluid; qPCR, quantitative polymerase chain reaction.

(a) Bloodstream infection: peripheral blood is the first choice for bloodstream infection detection. mNGS significantly improves the accuracy of bloodstream infection diagnosis, minimizes pathogen diagnosis time, and facilitates the rational use of antibacterial drugs (Li et al., 2021).

(b) Central nervous system infection: mNGS has a higher detection rate than conventional detection methods for bacterial infections of the central nervous system and is less affected by antibacterial drugs (Wilson et al., 2019; Zhang et al., 2019). Moreover, mNGS have irreplaceable advantages in the diagnosis of central nervous system tuberculosis, viruses, special pathogens (such as Brucella), parasites, and other pathogen infections, whereas fungal pathogens such as Cryptococcus and Aspergillus need to be detected in conjunction with traditional methods (Yao et al., 2016; Fan et al., 2018; Wang et al., 2018; Xing et al., 2020; Yan et al., 2020). Additionally, mNGS helps diagnose non-infectious disorders such as autoimmune encephalitis and aids in selecting appropriate medicines (Wilson et al., 2019). It is recommended to employ a combination of mNGS and traditional microorganism detection methods for the diagnosis of chronic or recurrent central nervous system infections. This combination of methods is also the second-line diagnostic approach for acute encephalitis cases (Li et al., 2021).

(c) Respiratory infection: for respiratory tract infections, mNGS enables rapid and accurate pathogen detection and identification. It has been shown that mNGS application improves the diagnosis of lung-invasive fungal infections (Li et al., 2018). The detection rate of fungal etiology in bronchoalveolar lavage fluid (BALF) mNGS samples (81.3%) was significantly greater than that in blood samples (25.0%) in pulmonary invasion fungal infection; however, the types of samples had a similar specificity (Chen et al., 2020). mNGS sensitivity did not differ substantially between respiratory specimens (Li et al., 2021), but BALF was more sensitive than sputum for the detection of nontuberculous mycobacteria (Miao et al., 2018). Moreover, mNGS outperforms existing detection methods in the identification of mixed lung infections. In immunocompromised patients, the detection accuracy rate of mNGS may even reach 100% (Li et al., 2020).

(d) Bone and joint infections: synovial fluid, ultrasonic cleaning fluid from implants, and periprosthetic tissue can be used as specimens for mNGS (Ivy et al., 2018; Huang et al., 2019). mNGS is particularly recommended for pathogen detection in patients with negative microbiological culture results, poor response to empirical antibiotic therapy, and inadequate debridement (Fang et al., 2020).

(e) Urinary tract infections (UTIs): mNGS is not superior to the traditional detection methods for bacterial and fungal UTIs; however, it has advantages in diagnosing viral and complex UTIs (Flores-Mireles et al., 2015). The limitation of mNGS in urine testing is that reporting interpretation is difficult, and colonization of the distal urethra, periurethral skin, and vagina interferes with and requires experienced clinicians to participate in the interpretation (Mouraviev and McDonald, 2018).

(f) Digestive system infection: mNGS is rarely used in the identification of intestinal pathogens, and its greater application value is reflected in the identification of drug resistance genes in intestinal pathogenic microorganisms as well as the identification of the causes of rare viral hepatitis (Kujiraoka et al., 2017; Somasekar et al., 2017).

(g) Complex and atypical pathogen infections: many cases have demonstrated the superiority of mNGS in identifying infections with complex and atypical pathogens (Li et al., 2021). Moreover, mNGS has shown significant advantages in pathogen identification in immunocompromised patients (Parize et al., 2017). It is recommended as a first-line infection diagnosis and surveillance tool or as a complementary means to routine testing in patients undergoing solid organ and hematopoietic stem cell transplantations (Carpenter et al., 2019). mNGS has considerably improved pathogen diagnosis in febrile patients and is expected to be utilized as an integrated diagnostic tool for patients with fever of unknown origin to discriminate between infectious and non-infectious origins as well as to detect emerging pathogens (Jerome et al., 2019). Furthermore, mNGS helps monitor and respond to rapidly evolving infectious diseases such as unexplained pneumonia, e.g., COVID-19 (Duan et al., 2021).

Sample types, such as blood, sputum, BALF, cerebrospinal fluid (CSF), pleural fluid, pus, and tissue specimens, can be used in mNGS (Duan et al., 2021). Specific samples must first be collected from the primary site of infection for the diagnosis of infectious diseases—for instance, CSF is typically advised for infections of the central nervous system, whereas BALF and sputum are generally advised for infections of the lungs. Even though library creation, sequencing, and bioinformatics analysis are the same for all samples, pretreatment varies depending on the source of the sample (Li et al., 2021). Sputum requires liquefaction treatment, FFPE samples require dewaxing, and tissue requires homogenization. For specimens containing a large human cell background, such as secretions, pus, tissue specimens, and cloudy CSF and BALF, sequences need to be removed from the analysis by appropriate de-hosting processes to improve overall detection sensitivity (Charalampous et al., 2019). Using techniques such as filtering, differential centrifugation, DNA enzymatic hydrolysis, and methylation reagent treatment, the amount of human DNA in samples can be decreased (Li et al., 2021). Cell-free DNA or cell-free RNA extraction strategies are generally used in samples from peripheral blood (Charalampous et al., 2019). In bioinformatics analysis, human nucleic acid sequences are filtered first. The proportion of sequences removed by filtering steps was significantly correlated with tissue type, the abundance of infectious microorganisms, and/or the degree of environmental contamination (Ramachandran and Wilson, 2020). Specifically, 97–99% of the sequences in CSF samples from patients with encephalitis may be human because such samples are typically pathogen-free (Ramachandran and Wilson, 2020), whereas for sputum from patients with viral pneumonia infections, 80% of the sequences may be viral (Duncan M, 2019). Different threshold mechanisms must be created for different types of samples and pathogens for downstream analysis (Li et al., 2021). Notably, mNGS results need to be combined with clinical presentation—for example, when the mucormycosis pathogen is detected in peripheral blood, bloodstream or local infections should be judged according to the clinical manifestation and image due to the fact that *Mucor* DNA fragments can easily enter the blood during a local infection (Jing et al., 2021).

Early antifungal management, proper surgery, and immunosuppression reversal are all essential components of mucormycosis treatment (Phoompoung and Luong, 2019). The latest guidelines recommend liposomal amphotericin B as the first-line therapy (Phoompoung and Luong, 2019). In the first case that we presented herein, although AmBL (1 mg/kg daily, IV) was administered immediately after mucormycosis had been diagnosed, the local symptoms did not improve, and fever returned 3 days later. This was likely because the recommended dosage of AmBL for mucormycosis treatment is 3–5 mg/kg/day. Due to the toxicity of AmBL, moderate escalation is required; effective plasma concentrations cannot be attained quickly, resulting in therapy failure. A patient’s general conditions can improve significantly after the administration of ABCD (3.3 mg/kg daily, IV).

## Conclusion

We presented two cases of patients with lymphoma who developed severe nasal and cutaneous mucormycosis following chemotherapy and small molecule targeted therapy. Traditional laboratory tests and imaging failed to determine the possible infection etiology; however, mNGS was able to rapidly and accurately determine the pathogens, and the results were later validated in local secretory fungal cultures. Both patients improved rapidly after receiving ABCD antibiotic treatment and experienced a good clinical outcome.

Our cases demonstrate that mNGS is a sensitive, efficient, accurate, and straightforward diagnostic technique that improves diagnostic accuracy and treatment efficacy. It is reasonable to predict that, as sequencing prices fall, mNGS will become widely used as a clinical pathogen detection approach.

## Ethics statement

Written informed consent was obtained from the individual for the publication of any potentially identifiable images or data included in this article.

## Author contributions

QZ, XL, YL, HW, RZ, XW, and KZ were all involved in the clinical care and management of the patient. XLv performed the laboratory and mNGS testing and data interpretation. QZ wrote the initial draft of the manuscript and did the literature search, figures, and data collection. QZ wrote the final manuscript—review and editing. KZ and XW did the data interpretation and wrote the final manuscript—review and editing. All authors contributed to the article and approved the submitted version.

## Funding

This work was supported by grants from the NationalNatural Science Foundation of China (81470336) and ChinaInternational Medical Foundation(Z-2018-35-2003).

## Conflict of interest

The authors declare that the research was conducted in the absence of any commercial or financial relationships that could be construed as a potential conflict of interest.

## Publisher’s note

All claims expressed in this article are solely those of the authors and do not necessarily represent those of their affiliated organizations, or those of the publisher, the editors and the reviewers. Any product that may be evaluated in this article, or claim that may be made by its manufacturer, is not guaranteed or endorsed by the publisher.
